# Croup

**Published:** 1855-04

**Authors:** 


*Croup.*—The American Medical Monthly for August and September, contains a long and interesting paper by Professor E. R. Peaslee, on *The Pathology of Croup, as the Basis of its Rational Treatment.* The article gives evidence of much thought and research on the part of its author, and we are sorry that we can do no more than give a mere outline of his ideas of the pathology of the disease and its treatment. This we do in his own words:

1. An inflammation of the larynx, extending into the trachea, occurs; offering in its essential nature nothing different from any other case of inflammation of the same parts, either in the infant or the adult. It is generally preceded, in both infants and adults, by congestion and irritation, and therefore by catarrh.

2. An exudation of plasma occurs on the inflamed surface, as in the adult; this being most abundant, in the trachea, on the posterior wall.

3. This exudation may be disposed of in at least two ways, provided it is not at once removed, as it generally is, in adults but not in infants, by coughing; reäbsorption probably very seldom occurring in this disease, though it is not impossible.

*a.* It may become degenerated into pus (purulent matter,) and thus, of course, at once be detached, which is the most common result.

*b.* It may become organized into a false membrane. This is more probable if the blood is rich in fibrin (*e. g.* in a plethoric child); if there is but little cough (an adult generally expelling it thus,) and if time is allowed for its development (less being required in the child than in the adult.)

4. Croup is, therefore, merely a laryngo-tracheitis in infants and children, and offers nothing essentially different from the same inflammation in adults.* The exudation in case of adults is, however, usually at once ejected by coughing, or in the form of purulent matter; while the liability to its organization in infants is greater; though, after all, a comparatively rare result, considering the whole number of cases—for the reasons before mentioned.†

5. Practically, therefore, as well as pathologically, we cannot say with Bouchut: "Without a false membrane croup does not exist." This membrane never exists till the inflammation—the essential element of the disease as we believe—has preceded, and has produced the exudation of plasma, as before shown. No sooner does the catarrhal *irritation* merge into inflammation, than the plastic lymph is thrown out;‡ and this *inflammation and its accompanying exudation* are the elements always present in croup.§

We therefore need not, for any practical purpose, admit an "inflammatory and a membranous" croup, as some writers have done, any more than we should make the same distinction in regard to pleuritis or peritonitis. All croup is *inflammatory*, at any rate; and a few cases are also accompanied by the formation of a false membrane. But the latter should not affect the treatment of the disease as an inflammation, but merely from its mechanical effects, and cannot be predicated in any case till it is actually seen; and this is not possible in most cases in which it is developed at the very onset of the disease.

---

* The idea of Copland and others, that the false membrane in infants is due to a greater amount of *albumen* in the blood, is entirely without support.

† In Dr. Ware's "Contributions to the History and diagnosis of Croup," false membrane was found in only twenty-two cases out of 131, or about one-sixth of the whole.

‡ Hasse's Diseases of Organs of Circulation and Respiration, p. 277.

§ Dr. H. Green also holds this idea, though in different terms. See his work on Croup, p. 13.

Finally, we would drop the term croup entirely, and use the term laryngo-tracheitis instead. In a work on the diseases of children, we would call particular attention to the fact that a false membrane is formed in about one-sixth of all the cases of this disease; while in adults, this is of very rare occurrence. But we would not make an accident the distinguishing feature of this disease, more than we do in the case of others, nor allow it to enter into either our name or our definition of it.

As in all other inflammations, so in this; the distinction of "sthenic" and "asthenic" is important, both in a pathological and a therapeutical point of view. So far also as laryngismus enters into any particular case—and it does into all cases of true laryngitis to some extent—the case is, of course, *spasmodic;* but this term must not be applied to the exclusion of the idea of inflammation. *Genuine spasmodic* croup, we have already seen, is a mere laryngismus. There is more or less spasm in all cases of bronchitis, and still more in whooping-cough; in the latter case in the larynx also; so that infantile laryngitis does not present any peculiarity in this respect. *Catarrhal* infantile *laryngitis* we regard as a contradiction of terms.

The treatment he recommends for the two forms of the disease is presented in the following tabular form:

| | *Sthenic Laryngo-tracheitis.* | *Asthenic Laryngo-tracheitis.* |
|---|---|---|
| I. To arrest the inflammatory process, and control the laryngismus. | An emetic, followed by a full dose of calomel, if required. | Do. do.; or pil. hydrargyri, instead of calomel. |
| | Leeches to throat, or over the sternum; followed by cold applied continuously. | Leeches doubtful. Cold continuously applied. |
| | Tartrate of antimony; sedative doses, cautiously administered. | Nitrate of potassa. |
| | Internal application of solution of nitrate of silver. | Do. do. |
| | Inhalation of aqueous and narcotic vapors. | Do. do. |
| | Opiates, with caution. Hydrocyanic acid, do. do. | Do. do. |
| II. To prevent organization of the exuded plasma. | Sponge-probang and caustic solution. | Do. do. |
| | Expectorants. | Stimulating expectorants |
| | Calomel, in small doses. | Turpeth mineral. |
| | Alkalies. | Do. |
| | Counter-irritants. | Do. |
| | Tracheotomy? | |
| III. For removing the false membrane when formed. | Continue caustic solution | Do. do. |
| | Do. expectorants. | Do. |
| | Do. counter-irritation. | Do. |
| | Do. alkalies. | Stimulant alkalies. |
| | No emetics, or calomel. | Do. do. |
| | Tracheotomy, *seasonably,* if at all. | Do. do. |
| IV. To sustain the strength. | Milk-porridge, arrow-root, broth, &c. | Do. do.; also wine whey, milk-punch, &c. |
| | Tonics, if required. | Tonics and stimulants. |

—*American Medical Monthly* from *New Jersey Medical Reporter.*

